# Neuroendocrine Tumors: Genomics and Molecular Biomarkers with a Focus on Metastatic Disease

**DOI:** 10.3390/cancers15082249

**Published:** 2023-04-12

**Authors:** Erica S. Alexander, Etay Ziv

**Affiliations:** Department of Radiology, Memorial Sloan Kettering Cancer Center, New York, NY 10065, USA; alexane@mskcc.org

**Keywords:** neuroendocrine tumors, Ki-67, chromogranin A, 5-HIAA, NETest, ablation, embolization

## Abstract

**Simple Summary:**

Liver metastases secondary to neuroendocrine tumors (NETs) are common at the time of diagnosis and are associated with worse quality of life and overall survival. This review focuses on the biomarkers and genetics associated with NETs, with a particular focus on disease diagnosis, prognosis, and treatment options. Specifically, liver-directed therapies are highlighted as meaningful treatment options for liver-dominant NETs.

**Abstract:**

Neuroendocrine tumors (NETs) are considered rare tumors that originate from specialized endocrine cells. Patients often present with metastatic disease at the time of diagnosis, which negatively impacts their quality of life and overall survival. An understanding of the genetic mutations that drive these tumors and the biomarkers used to detect new NET cases is important to identify patients at an earlier disease stage. Elevations in CgA, synaptophysin, and 5-HIAA are most commonly used to identify NETs and assess prognosis; however, new advances in whole genome sequencing and multigenomic blood assays have allowed for a greater understanding of the drivers of NETs and more sensitive and specific tests to diagnose tumors and assess disease response. Treating NET liver metastases is important in managing hormonal or carcinoid symptoms and is imperative to improve patient survival. Treatment for liver-dominant disease is varied; delineating biomarkers that may predict response will allow for better patient stratification.

## 1. Introduction

**Figure 1:**
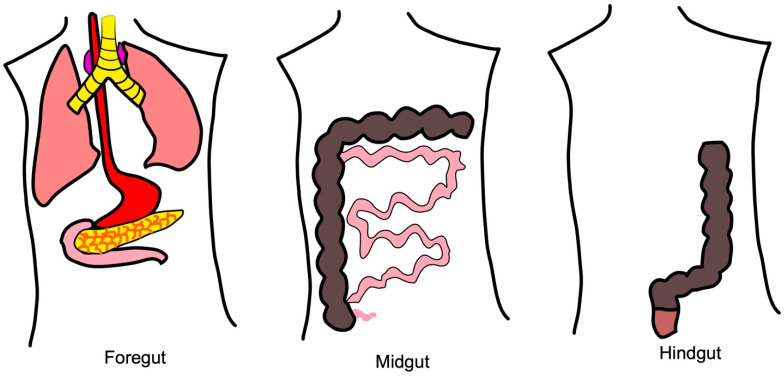
Williams and Sandler provided one of the earlier NET classifications still used today. They divided tumors according to their embryologic site of origin: foregut carcinoids are those arising from the respiratory tract, stomach, duodenum, biliary system, and pancreas; midgut carcinoids arise from the small intestine, appendix, cecum, and proximal colon; and hindgut carcinoids arise from the distal colon and rectum [2].

Neuroendocrine tumors (NETs) originate from specialized cells in the endocrine system and are associated with symptoms secondary to the release of vasoactive peptides and hormones. They most commonly originate in the gastrointestinal (GI) tract but can also occur in the lung, pancreas, and other organs [1]. NETs represent a heterogeneous array of malignancies and have been described and subdivided using many classification systems. The most commonly used classifications are based on the location of origin, originally described by Williams and Sandler (Figure 1); release of hormones resulting in clinical symptoms; tumor grade; and rate of division and growth (Table 1) [2,3]. It should be noted that NETs are distinct entities from poorly differentiated neuroendocrine carcinomas and mixed-histology neuroendocrine neoplasms.

Often considered a rare cancer, the incidence of NET diagnosis in the United States has increased over the past several decades [5,6]. Many NET patients present with metastatic disease at the time of diagnosis [7]. Unsurprisingly, histologic differentiation and proliferative activity are strongly associated with the presence of metastatic disease. Pancreatic, right colon, and small intestine NETs are most associated with liver metastases at the time of diagnosis. In fact, in specialized tertiary centers 80–90% of patients with small-intestine NETs present with liver metastases [7]. The presence of metastatic NETs impacts both wellbeing and survival. NET liver metastases can cause debilitating symptoms, termed carcinoid syndrome, characterized by flushing, tachycardia, diarrhea, and bronchospasm [8,9]. Additionally, NET patients with liver metastases can develop biliary obstruction or hepatic insufficiency [10]. Survival is also impacted by the presence of liver metastases. Yao et al. found that patients with localized jejunal or ileal NETs had a median survival of 111 months, whereas those with metastatic disease had a median survival of 56 months [5].

The negative effect on quality of life and survival caused by metastases in NET patients highlights the necessity of better understanding the genetic makeup of these tumors—this understanding can allow for earlier disease detection, improved predictions, and expand treatment options.

## 2. Genomics

### 2.1. Inherited Disorders

Several inherited syndromes are associated with the development of NETs. Two of these syndromes, multiple endocrine neoplasia (MEN) types 1 and 2, are major contributors to the development of benign and malignant endocrine tumors. An additional six inherited syndromes have been associated with endocrine neoplasia: Carney complex, hyperparathyroidism–jaw tumor syndrome, von Hippel–Lindau, neurofibromatosis type 1, tuberous sclerosis, and familial isolated paraganglioma–pheochromocytoma syndrome [11,12]. These syndromes are overall quite rare and account for only a small portion of NET diagnoses. Notably, these genetic disorders are associated with well-studied mutations in tumor suppressor genes and oncogenes, which has helped drive increased knowledge about NETs at large.

#### 2.1.1. MEN Type 1

MEN type 1, also referred to as Werner syndrome, is a rare autosomal-dominant disorder characterized by the development of NETs in the pancreas, pituitary, and parathyroid glands, although tumors in other locations can occur [13]. The MEN1 gene on chromosome 11 controls production of menin, which possesses a tumor-suppressive function [14]. The lack of menin activity seen in MEN type 1 results in downregulation of p27Kipl and p181nk4c and, in turn, uncontrolled cell growth [12]. 

#### 2.1.2. MEN Type 2

MEN type 2 is a rare autosomal-dominant disorder characterized by tumors of endocrine organs. It is caused by defects of the rearranged during transfection (RET) proto-oncogene on chromosome 10, which in turn promotes cell growth, proliferation, and differentiation [15]. In general, MEN2A results in mutations affecting cysteine (634) and MEN2B results in the M918T mutation [12]. The classic presentation of MEN type 2 includes development of medullary thyroid carcinoma, pheochromocytoma, and primary hyperparathyroidism [16]. De novo mutations can occur in MEN2, particularly in MEN2B [16,17]. Understanding the genetic alterations that drive diseases such as MEN type 2 allows for possible treatment with RET inhibitors [16].

### 2.2. Sporadic Mutations

#### 2.2.1. Molecular Pathways of Foregut NETs

The sporadic mutations driving development of foregut NETs are best understood. Somatic deletion of the MEN1 gene has been found in a subset of sporadic foregut NETs. Loss of heterozygosity of 11q13 and mutations of the MEN1 gene have been described in up to 78% of sporadic NETs, with the highest frequency occurring with bronchial and pancreatic neuroendocrine tumors [13,18,19]. Loss of chromosome 3p is the most frequent change seen in lung NETs [11,20]. In lung NETs, loss of heterozygosity has also been associated with alterations involving chromosomes 5q21, 9p, 11q13, 13q13, and 17p13 [11]. Mutations in p53 and 5q21 in lung NETs are associated with aggressive tumors and poor survival [11].

Gastric NETS arise from enterochromaffin-like cells (ECLs); they are referred to as ECL types I-III and neuroendocrine carcinoma. ECL-I tumors account for >70% of all gastric NETs and present as multiple small tumors limited to the mucosa and/or submucosa; these tumors rarely metastasize. ECL-II tumors are associated with MEN type 1 and Zollinger–Ellison syndrome; these present as multiple tumors that infiltrate the submucosa. ECL-III tumors often result in hypergastrinemia and tend to present as a single tumor that infiltrates the muscularis propria and serosa layers and, in turn, frequently metastasizes [11,21]. Neuroendocrine carcinoma usually presents as a single large, poorly differentiated, and aggressive tumor. For neuroendocrine carcinoma, loss of heterozygosity has been reported on chromosomes 8p, 11p, 12p, 13q, 15q, and 17p [22].

In 2011, Jiao and colleagues studied the exomes of multiple pancreatic neuroendocrine tumors (PNETs) and screened for the most commonly mutated genes. Mutations in the MEN1, death-domain-associated protein (DAXX), and α thalassemia/mental retardation syndrome X-linked (ATRX) genes were found in 44.1%, 25%, and 17.6% of patients, respectively. The researchers also found mutations in the mammalian target of rapamycin (mTOR) pathway in 14% of tumors [23]. Several years later, Scarpa et al. performed whole-genome sequencing on a larger group of PNETs and discovered a deficiency in G:C>T:A base excision repair due to an inactivation of MUTYH. They also discovered that sporadic PNETs had a larger proportion of germline mutations [24]. Alterations in the P53 pathway, which normally regulates tumor suppression, have also been implicated in a high percentage of PNETs. Specifically, aberrations in the proteins that regulate the p53 pathway, including MDM2, MDM4, and W1P1, are thought to play an important role in the initiation and progression of PNETs [25]. Abnormal immunolabeling of p53 has been detected in poorly differentiated PNETs, but notably absent in well-differentiated PNETs. Additionally, retinoblastoma protein (Rb) expression is lost in a high proportion of poorly differentiated PNETs [26].

#### 2.2.2. Molecular Pathways of Midgut NETs

The molecular pathways driving midgut NETs are less understood; however, comparative genomic hybridization studies have demonstrated loss of chromosomes 9p, 11q, 16q, 18p, and 18q in small bowel carcinoid tumors [27,28,29,30,31,32]. The allelic loss of chromosome 18 was, in one study, present in 69% of ileal carcinoid tumors [27]. Mutations of chromosome 11q, affecting the tumor suppressor gene succinate dehydrogenase complex subunit D (SDHD), have also been associated with midgut NETs [30]. Mutations in cyclin dependent kinase inhibitors (CDKNs) have been associated with midgut-derived NETs, with focal deletion peaks at 9p21 (CDKN2A/B) and 12p13 (CDKN1B) [33]. Interestingly, Kytola and colleagues found that the number of alterations were significantly higher in metastases compared to primary tumors, suggesting an accumulation of acquired genetic changes as tumors progressed. Both primary and metastatic tumors showed losses in 18q and 11q, whereas losses in 16q and 4 were only detected in metastatic disease [28].

#### 2.2.3. Molecular Pathways of Hindgut NETs

There is limited information on the genomics of hindgut NETs relative to foregut and midgut NETs [11]. Shimizu et al. studied the growth characteristics of rectal carcinoid tumors and found that rectal carcinoids larger than 5 mm have significantly higher levels of Ki-67 and more frequent expression of TGF-alpha [34]. Watanabe’s group found that there was a significant correlation between Ki-67 levels and glucose transporter 2 (GLUT2) scores and O_6_-methylguanine DNA methyltransferase (MGMT) scores in hindgut NETs [35]. Overall, hindgut NETs are thought to develop via different molecular pathways compared to foregut and midgut NETs.

## 3. Diagnostic Biomarkers

The North American Neuroendocrine Tumor Society (NANETS) consensus guidelines provide recommendations for biomarkers as they relate to surveillance and prognosis of disease. 

The biomarkers and peptide hormones produced and secreted by NETs can result in the pathognomonic syndromes of functional neuroendocrine tumors. Associations are well-established between urinary 5-HIAA levels and carcinoid syndrome, serum or plasma gastrin and Zollinger–Ellison syndrome, insulin and insulinomas, and glucagon and glucagonomas, along with other functional syndromes [36]. For this section, we will discuss those biomarkers most evaluated for diagnosing and surveilling NETs. Notably, there is much controversy about the utility of biomarkers in diagnosing NETs, as tissue sampling is currently the most sensitive way to diagnose these malignancies. 

### 3.1. Chromogranin A

Chromogranin A (CgA) is a 49-kDa protein present in the neurosecretory vesicles of NET cells; it is a very specific marker for patients with endocrine neoplasms and can be detected in blood plasma and serum samples, as well as from tissue immunostaining [36]. CgA is a widely used biomarker in NETs and is co-secreted with the amines and peptides located in the neurosecretory granules [37,38]. CgA levels are influenced by tumor cell type and histologic differentiation [37]. For example, CgA is elevated in patients with gastrinoma and NETs of midgut origin when compared to disease-free controls [39,40]. Elevations in CgA levels have been shown to correlate with tumor burden, presence of metastases, and response to treatment [41,42]. One limitation of measuring CgA levels is that they can be elevated due to other conditions/physiologic changes, including heart disease, gastric acid suppression, pulmonary disease, rheumatologic conditions, renal dysfunction, food intake, and other cancer types [41]. 

### 3.2. Synaptophysin

Synaptophysin is a membrane glycoprotein of presynaptic vesicles and a useful biomarker for NETs or neural tissues. Synaptophysin has been found in paragangliomas, pheochromocytomas, thyroid medullary carcinomas, and pancreatic islet cell tumors [43,44]. It is considered a very sensitive biomarker in the diagnosis of NETs and tends to be detectable in tumors with varying degrees of differentiation; it can be analyzed using immunohistochemistry [45]. A limitation of this biomarker is that it can be detected in other cancers, including pseudopapillary tumors of the pancreas, adrenocortical carcinoma, and non-small-cell lung cancers [46,47,48]. In Kriegsmann et al.’s retrospective review of 1170 tissue samples, synaptophysin was commonly expressed in pulmonary adenocarcinomas and squamous cell carcinomas [48]. 

### 3.3. 5-HIAA

In patients with metastatic midgut carcinoid tumors, particularly functional midgut NETs, 5-HIAA is generally elevated. 5-HIAA represents the primary metabolite of serotonin, and urinary 5-HIAA and serum and/or plasma 5-HIAA are proxies for serotonin measurement [49]. Urinary 5-HIAA is the primary clinical assay used to assess for midgut NETs, with a sensitivity and specificity up to 83% and 95%, respectively [50,51]. Several assays can be used to measure 24 h urine 5-HIAA, but high-performance liquid chromatography is most frequently used [52]. The 24 h urinary serotonin should be measured in patients for whom carcinoid syndrome is indeterminate, as these patients generally have consistent elevations in urinary 5-HIAA [10,37]. Janson et al. evaluated over 300 patients referred to a tertiary center for management of NETs. Among those patients, urinary 5-HIAA was increased in 76% of those with midgut NETs and 48% of those with bronchial carcinoid tumors. Additionally, those patients with midgut NETs and carcinoid heart disease had significantly higher levels of urinary 5-HIAA [53]. False elevations in urinary 5-HIAA can occur from ingestion of tryptophan/serotonin-rich foods and from certain medications and/or supplements, including acetaminophen, nicotine, and caffeine [54,55].

### 3.4. NETest

NETest is a newer multigenomic blood assay, comprised of 51 marker genes, which uses PCR technology and multianalyte algorithmic analyses to test for neuroendocrine neoplasms. The NETest utilizes four mathematic tools, including support vector machine, linear discriminant analysis, k-nearest neighbors, and the naïve Bayes algorithm to differentiate tumors from controls and to differentiate stable disease from progressive disease [56]. Additionally, gene expression profiling and machine learning have been shown to accurately predict the development of NET metastases [57].

Circulating biomarkers from NETs can be assayed using a liquid biopsy. The major advantages of the NETest are that it can be used to identify all types of NETs and it can detect early-stage nonmetastatic disease [56,58]. Several studies have validated the NETest’s sensitivity and specificity in identifying NETs and detecting early disease recurrence [59,60,61]. The NETest provides an “NET activity score,” which ranges from 0–100: values > 20 are abnormal, stable disease is between 21–40, and progressive disease is 41–100 [62]. The NETest is significantly more accurate than CgA assays in differentiating NETs from controls and in detecting disease progression [60,61].

### 3.5. Somatostatin Receptor Imaging

Somatostatin receptors (SSRs) are present on the cell surface of neuroendocrine cells; when somatostatin binds to the SSR it exerts an effect on neurotransmission, hormone secretion, and cell proliferation [63]. SSRs provide a specific molecular target for somatostatin receptor (SSR) imaging. ^111^In-pentetreotide represents the earliest success of SSR imaging and was pivotal in localizing gastroenteropancreatic NETs and glomus paragangliomas [64]. ^68^Ga-DOTATATE positron emission tomography–computed tomography (PET/CT) is currently the molecular imaging modality of choice for imaging and localizing NETs, with a sensitivity greater than 90% and a specificity ranging from 92–98% [65,66,67]. ^68^Ga-DOTATATE PET/CT has an important role in the diagnosis and management of NETs by confirming the diagnosis of NET, localizing unknown primary NETs, and by identifying additional sites of disease that can stratify surgical versus nonsurgical patients [63]. Notably, standardized uptake values on ^68^Ga-DOTATATE PET have significant correlation with SSR density on histologic specimens [68].

## 4. Prognostic Biomarkers and Genes

### 4.1. Ki-67 Proliferation Index

NETs represent a wide spectrum of malignancies, from indolent, well-differentiated tumors to more aggressive, poorly differentiated tumors. Well-differentiated NETs are divided into three grades (G1–G3) based on Ki-67 proliferation. The Ki-67 index is calculated by either visually counting the cells within the field of maximum staining or via automated digital analysis of the field of view using computer software. G1 tumors are defined by Ki67 < 3%, G2 tumors are defined by Ki67 between 3–20%, and G3 are defined by Ki67 > 20% [69]. In general, high-grade tumors, those with a mitotic count of more than 20 per 10 high-powered fields, or with a Ki-67 proliferation index greater than 20%, represent aggressive malignancies [70]. Hamilton et al. stained pathologic specimens of patients who underwent pancreatic resection for PNET and found that patients with a Ki-67% > 9% were significantly more likely to have disease recurrence and decreased overall survival compared to those patients with lower values [71]. Response to medical therapy, including somatostatin analogs, has also been found to correlate with Ki-67 levels [72,73]. The prognostic value of Ki-67 in outcomes for liver-directed therapy has also been described. Chen et al. found in their multicenter retrospective study that a higher tumor grade resulted in significantly shorter hepatic progression-free survival and overall survival [74]. Comparison of treatment outcomes between different embolic therapies and Ki-67 scores has also been performed. A single-institution retrospective study found that in NET patients with a Ki-67 score ≥ 3%, yttrium-90 transarterial radioembolization (Y90 TARE) was associated with a better overall survival when compared to transarterial chemoembolization (TACE); however, the inverse was true in patients with a Ki67 < 3% [75]. G3 NETs have been divided into two distinct classes of tumors: well-differentiated NETs with low proliferative capacity and poorly differentiated neuroendocrine carcinomas. The latter have distinct morphologic features, present with distant metastases, and have worse prognosis [76]. Notably, well-differentiated NETs have intact p53 and demonstrate no Rb loss in immunohistochemistry.

### 4.2. Chromogranin A

High levels of plasma CgA portends a worse prognosis in patients with NETs [36,53,77]. Janson et al. evaluated 301 patients with NETs and discovered that plasma chromogranin A > 5000 micrograms/l was an independent predictor of poor survival. Those patients with elevated plasma CgA > 5000 micrograms/l had a median survival of 33 months compared to 57 months in patients with lower CgA values [53]. Several studies have shown that decreases in CgA levels after initiation of therapy are associated with positive treatment response [78,79,80,81]. Reduction or normalization of CgA has been described in tumors treated with liver-directed treatments, including radio-frequency ablation and embolization [80,81].

### 4.3. 5-HIAA

In patients with midgut NETs, 5-HIAA levels have been shown to predict the presence of liver metastases, disease progression, and survival [53,82]. In a prospective single-institution study, Wedin et al. found that in patients with well-differentiated NETs, the presence of liver metastases and/or extensive liver involvement (>10 metastases or diffuse metastatic disease) were associated with significantly higher levels of serum 5-HIAA levels [82]. Researchers have shown that patients with urinary 5-HIAA > 300 μmol/24 h have significantly shorter median survival compared to patients with lower levels [53]. Additionally, the rate of increase of urinary 5-HIAA is predictive of prognosis. Patients with small-intestine NETs or NETs of unknown primary with a urinary 5-HIAA doubling time < 434 days had a significantly higher disease-specific mortality compared to those with longer doubling times [83]. Several studies have described reduction of 5-HIAA levels after transarterial liver-directed therapy [84,85,86,87]. Carrasco et al.’s study evaluating hepatic artery embolization in patients with malignant carcinoid syndrome found reduction in urinary 5-HIAA levels in all patients who had objective imaging and clinical response [86]. When combined with systemic therapy such as interferon therapy, hepatic artery embolization has been shown to have greater effect in controlling carcinoid symptoms and decreasing 5-HIAA levels compared to interferon therapy alone [87]. Thermal ablation therapy has also been found to improve carcinoid symptoms and decrease 5-HIAA levels [88].

### 4.4. DAXX or ATRX Mutations

Exome sequencing of sporadic pancreatic NETs has shown that nearly half of these tumors have somatic mutations of the DAXX or ATRX genes [23]. DAXX and ATRX mutations are mutually exclusive, but their encoded proteins are thought to function in the same pathway.

The wild-type DAXX/ATRX complex regulates chromatin remodeling; however, the impact of DAXX/ATRX loss on tumor progression is poorly understood [89]. DAXX and ATRX mutations in PNETs have paradoxically been described to have both positive and negative effects on prognosis [23,90]. Cives and colleagues evaluated genomic variations in patients with pancreatic NETs. The authors found that larger tumors (>2 cm) were significantly more likely to have DAXX mutations. DAXX mutations were, in turn, associated with higher disease grade, nodal involvement, lymphovascular invasion, and were predictive of relapse after surgery and decreased disease-free survival [91]. On the other hand, Jiao et al. found that mutations in the DAXX or ARTX genes were associated with improved prognosis compared to wild-type PNETs [23]. This difference may be related to the different cohorts of patients in these studies. For example, specimens in Cives’ group were largely taken from primary sites during surgery. The impact of DAXX mutation status on outcomes of liver-directed disease has also been evaluated. Ziv et al. reported outcomes of 51 patients with NET liver metastases who underwent transarterial embolization; those patients with DAXX-mutant PNETs had shorter hepatic progression-free survival and shorter time to hepatic progression [92].

## 5. Biomarkers Associated with Treatment Response

Treatment for NETs remains diverse; options include somatostatin receptor agonist blockade, targeted radionuclides, chemotherapy, targeted therapies, liver-directed therapies, and surgery [50,93]. The diverse treatment options available highlight the need to match patients, specifically their genetic alterations and biomarker expressions, to appropriate treatment options. One of the major limitations in current treatment strategies for metastatic NETs is that there is a very limited understanding of predictive biomarkers. The field ultimately requires a better understanding of how genes or molecular markers impact treatment-specific outcomes.

### 5.1. Somatostatin Synthetic Analogs

Somatostatin synthetic analogs (SSAs) were initially introduced in 1987 for the management of carcinoid syndrome [94]. Around eighty percent of gastroenteropancreatic NETs express somatostatin receptors (SSR) [95]. By binding to somatostatin receptors, specifically somatostatin receptor 2, SSAs suppress excess hormone secretion and cell proliferation and can allow for stability or shrinkage of tumors. SSAs are mainstays in treating both functional and nonfunctional NETs and are generally used as the frontline antitumor therapy in patients with NETs [96]. Both the PROMID and CLARINET trials showed increased efficacy of SSA therapy in patients with disease that was positive on somatostatin receptor imaging [73,97]. Predictive biomarkers for poor response to SSAs include grade 2 tumors (as defined by Ki-67 index) and levels of serum CgA greater than ten times the upper limit of normal [98]. Butturini et al. also reinforced this finding; their study showed that the Ki-67 proliferative index was a significant predictor of response to SSAs; patients with Ki-57 ≥ 5% had significantly greater rates of tumor progression [99].

### 5.2. Somatostatin Receptor Imaging

Somatostatin receptor (SSR) imaging enables detection of receptor-positive neuroendocrine tumors, which in turn allows for consideration of peptide receptor radionuclide therapy (PRRT). A joint consensus statement by NANETS and the Society of Nuclear Medicine and Molecular Imaging (SNMMI) states that ^177^Lu-DOTATATE PRRT should only be used to treat patients with SSR-positive tumors [100].

Several studies have shown that increased uptake on SSR imaging correlates with better long-term prognosis when treated with PRRT [101,102]. Kratochwil et al. proposed that a tumor-to-liver SUVmax ratio greater than 2.2 may allow for better selection of patients who will respond to PRRT [102]. Other predictors of PRRT response include tumor grade and Ki-67 values. Studies have shown that patients with grade 3 NETs have an increased risk of disease progression and mortality compared to grade 1 and 2 patients after PRRT treatment [103,104,105]. In a large single-center study evaluating PRRT in 782 patients, Aalbersberg et al. found that Ki-67 > 5% resulted in lower progression-free survival and Ki-67 > 10% resulted in lower overall survival [106]. Baseline neuron-specific enolase concentration greater than 15 ng/mL has also been shown to have a negative impact on overall survival [107]. Bodei et al. studied predictors of efficacy of PRRT using circulating NET transcripts and CgA. They used the NETest to evaluate gene expression and found that patients who responded to PRRT had significantly higher growth factor signaling and metabolomic gene expression compared to non-responders [108].

### 5.3. Cytotoxic Chemotherapy

Cytotoxic chemotherapy is considered standard for patients with poorly differentiated NETs, but for patients with well-differentiated NETs its role is less defined. The primary types of chemotherapy used to treat NETs include kinase inhibitors, alkylating agents, and platinum agent-based combinations.

#### 5.3.1. mTOR Inhibitors

Mutations in the mTOR pathway, found in PNETs, represent a potentially treatable mutation. mTOR is a cytoplasmic protein kinase that helps regulate cell metabolism, growth, proliferation, and angiogenesis [109]. The mTOR inhibitor everolimus received FDA approval for the treatment of advanced PNETs in 2011 [110]. The approval was based on phase III data from the Radiant-3 trial, which showed that everolimus significantly prolonged progression-free survival in advanced PNETs compared to placebo [111]. RADIANT-4, a subsequent phase III trial, randomized patients with non-functional NETs of lung or gastrointestinal origin to receive either everolimus or placebo. Those patients treated with everolimus had significantly improved progression-free survival and a 52% reduction in the estimated risk of progression or death [112]. The results of this trial led to the expanded use of everolimus across NETs of different origin. In fact, the 2018 National Comprehensive Cancer Network (NCCN) guidelines describe everolimus as a recommended treatment option for patients with progressive metastatic gastrointestinal and bronchopulmonary NETs or for intermediate-grade bronchopulmonary NETs [113].

Only a subset of patients who receive mTOR inhibitors benefit from treatment; a greater understanding of biomarkers that predict response to everolimus remains crucial to best stratify patients. Benslama et al. evaluated the relation between phosphorylated p70S6K expression and everolimus response. This biomarker, a direct effector of the activated mTOR complex, was chosen by the authors due to the availability of a robust immunohistochemical test that could be applied to archival tissues and because it had been validated in prior studies. They found that patients with higher levels of phosphorylated p70S6K had shorter progression-free survival when treated with everolimus compared to those patients with lower levels [114]. The MD Anderson group evaluated how treatment of PNETs with everolimus affected CgA and neuron-specific enolase levels. The authors found that elevated baseline CgA and NSE were associated with significantly shorter progression-free survival and overall survival [115].

#### 5.3.2. Alkylating Agents

The alkylating agents streptozocin, dacarbazine, and temozolomide are some of the most-used chemotherapy drugs for neuroendocrine tumors. These alkylating agents induce cell death through DNA damage caused by alkylating the O_6_-guanine site of DNA, which in turn causes DNA mismatch. One way that tumors overcome and develop resistance to these drugs is by increasing the expression of the DNA repair enzyme MGMT, which in turn restores DNA to its normal form [116]. Some studies have shown that low levels of tumor MGMT are associated with a positive response to alkylating agents, whereas high expression of MGMT minimizes chemotherapy-induced tumor death. PNETs can be associated with MGMT deficiency, which likely explains the greater sensitivity of PNETs to agents such as streptozocin and temozolomide, particularly when compared to carcinoid tumors of other origins [117]. In a small retrospective study, Hikioka et al. found that patients without MGMT expression had a significantly higher response rate to streptozocin when compared to patients who were MGMT-positive. The authors concluded that MGMT expression could be a potential biomarker for treatment of PNETs with streptozocin. Not all studies have supported using MGMT as a predictor of PNET response to alkylating agents [118,119]. Lemelin and colleagues are currently recruiting for a multi-institutional study across France to evaluate the relationship between MGMT status and alkylating agent response [120].

Combinations of alkylating agents with other chemotherapy drugs have also been studied and shown to have promising results [121,122,123]. Strosberg et al. retrospectively evaluated combined capecitabine and temozolomide in PNETs and found an objective radiographic response in 70% of patients and a median progression-free survival of 18 months. The authors found biochemical responses in many patients post treatment; 91% of patients with elevated CgA levels before beginning chemotherapy had normalization or >50% reduction in CgA levels after treatment with temozolomide and capecitabine [121]. The combination of capecitabine and temozolomide is thought to be synergistic because fluoropyrimidines, such as capecitabine, are thought to deplete MGMT, which ordinarily would allow for repair of the DNA damage created by temozolomide [124]. Capecitabine and temozolomide are also known radiosensitizers. Soulen et al. hypothesized that combining capecitabine and temozolomide with Y90 TARE would have added benefit for patients with liver-dominant NETs. The group found that combination chemotherapy and Y90 TARE resulted in a median reduction of the index lesion by 57%, with a mean hepatic progression-free survival of 42.5 months. Additionally, in the 20 patients with elevated CgA levels at baseline, there was an 87% median reduction of CgA levels after treatment [125].

Interestingly, alkylating agents have been found to produce continued tumor shrinkage in PNETs, even during treatment holidays. These treatment holidays are often initiated to reduce the risk of irreversible myelosuppression associated with alkylating agents. The NYP-Weill Cornell Medical Center and Memorial Sloan Kettering Cancer Center groups found that 72% of patients who underwent a treatment break had continued disease stability or shrinkage for a median of 19 months and referred to these patients as “exceptional responders.” In exceptional responders with available next-generation sequencing data, 83% had mutations in MEN1/DAXX/ATRX [126].

#### 5.3.3. Platinum Chemotherapy

The use of platinum agents in treating NETs has been well-described. A combination of fluorouracil plus oxaliplatin (FOLFOX) is the platinum regimen with the greatest supportive evidence [124]. A multicenter study evaluating FOLFOX in advanced, well-differentiated digestive NETs showed partial treatment response in 30% of PNETs, in 12.5% of small intestine NETs, and in 38.5% of NETs of unknown origin. Interestingly, the authors found that patients with insulinomas had improved progression-free survival compared to other pancreatic NETs [127]. Several studies have shown that FOLFOX provides a good disease control rate; however, markers or predictors of response are still largely unknown [127,128,129].

### 5.4. Interventional Radiology

Liver-directed interventional radiology therapies play a large role in the treatment of metastatic NETs. Locoregional treatment of hepatic metastases can aid in reducing bulk symptoms and hormonal symptoms as well as treating hepatic progression.

#### 5.4.1. Ablation Therapy

Ablation therapy has long been used to treat one or few small, discrete liver lesions. The earliest experiences with ablation therapy for NETs were described in the surgical literature. Patients treated with laparoscopic radiofrequency ablation (RFA) had subjective symptom relief and long-term local tumor control [130,131]. In the past two decades, trends have shifted toward image-guided percutaneous ablation performed by interventional radiologists.

Ablation therapy has long been found to provide good local tumor control with relatively low morbidity. In a nine-year retrospective review, Perrodin et al. compared outcomes of surgical management and microwave ablation (MWA). MWA of NET liver metastases was associated with a significantly lower rate of minor and major complications compared to surgery. Notably, there was no difference in local recurrence or mean survival between the two groups [132]. In a meta-analysis reviewing the efficacy of RFA on NET liver metastases, Mohan et al. found that in patients with symptomatic NETs, 92% reported symptom improvement after ablation [133]. Huang et al. recently published their single-institution review of ultrasound-guided RFA in treating metastatic NETs. They reported a median progression-free survival of 15 months; for patients with a Ki-67 < 5%, the progression-free survival was significantly longer [134]. Several studies have also reported worse ablation outcomes in patients with elevated Ki-67 proliferative indices [88,135].

#### 5.4.2. Embolization Therapies

Embolization therapies, including bland embolization, chemoembolization, and radioembolization, have been used in the palliative management of metastatic NETs. Embolization is an accepted treatment, indicated for unresectable NET liver metastases in patients with bulk symptoms, excess hormone production, or disease progression. The hypervascular nature of NETs makes embolization-induced ischemia and/or targeted drug delivery an appealing treatment option.

Bland transarterial embolization (TAE) involves the delivery of small particles to the distal hepatic arteries supplying tumors. The occlusion of tumor vessels results in ischemia and tumor necrosis. Del Prete et al. reviewed the available literature on TAE and found that studies reported median survival rates between 10 to 80 months, with a progression-free survival as high as 60 months [136]. Several studies have shown that TAE is associated with reductions in 5-HIAA levels [85,86,137,138,139]. In fact, Sward and colleagues found that in patients who underwent TAE of NET liver metastases, decreases in urinary 5-HIAA and plasma CgA were predictors of survival [139].

Transarterial chemoembolization (TACE) is appealing due to the regional delivery of chemotherapy, allowing for increased intratumoral drug concentration and increased drug exposure time. Many studies have compared the efficacy of TAE vs. TACE for treatment of NET hepatic metastases, but results are largely equivocal [140]. Touloupas et al. recently published their 15-year experience treating NETs with TACE. In their review of over 200 patients, they found a median overall survival of 5.3 years; patients who demonstrated tumor response by RECIST or mRECIST had significantly longer median survivals compared to those who did not respond [141]. Few studies have evaluated biochemical responses after TACE treatment. A study from an Ohio State University group revealed that serum pancreastatin levels were significantly correlative with overall survival, with levels <9999 pg/mL being associated with longer survival [142].

Radioembolization is another treatment option for metastatic NETs. Y90 TARE uses glass or resin microspheres and is considered a microembolic therapy, relying on radiation-induced cytotoxicity to achieve cell death. In 2008, Rhee and colleagues published their preliminary multi-institutional results in treating NETs with resin or glass Y90. The median survival for patients treated with glass microspheres was 22 months, and the median survival for those treated with resin microspheres was 29 months (*p* > 0.05) [143]. Ten years later, Jia et al. published a systematic review of Y90 TARE for NET liver metastases. The authors reviewed 11 studies and 7 abstracts and found that the median disease control 3 months after treatment was 86% and the median survival was 28 months [144]. Knowledge of biomarkers that impact Y90 TARE outcomes is limited. Tsang et al. retrospectively reviewed Y90 TARE outcomes in 49 patients with liver-dominant NETs and found that low Ki-67 was a significant predictor of overall survival [145]. However, Singla et al.’s group found that patients with Ki-67 ≥ 3% who underwent Y90 TARE fared better compared to those who underwent TACE [75].

### 5.5. Surgical Treatment

In patients with metastatic NETs, NCCN guidelines support complete resection of the primary site of disease and metastatic sites when feasible [146]. Guidelines even suggest a role in cytoreductive surgery when 70% or more of the overall disease burden can be treated [147]. To date, hepatic resection offers the only potential curative option for patients with metastatic NETs, with 5-year survival rates quoted up to 80% [148]. Mayo et al. reviewed surgical outcomes of patients with NET liver metastases in a large multicenter international cohort. In this study the median survival was 125 months, with an overall 5-year survival of 74%. No biomarkers were evaluated; however, authors found that patients with nonfunctional NETs, synchronous disease, and extrahepatic disease had worse survival. The authors concluded that patients with hormonally functional liver metastases, without extrahepatic or synchronous disease, derive the best survival benefit from surgery [149]. A recent study published by Modlin et al. evaluated the use of NETest to predict surgical outcomes. Prior to surgery, NETest levels were elevated in all patients; after curative R0 surgery the NETest levels were normal in 70% of patients; after palliative R1/R2 surgery, all tests remained elevated. Additionally, NETest was significantly more accurate in detecting the progression of disease after surgery compared to CgA [59].

Hepatic NET metastases are often multifocal and bilateral at the time of diagnosis, making resection challenging. Several authors have described promising results when combining hepatectomy and ablation therapy for treatment of metastatic NETs [135,150,151]. Taner et al. retrospectively analyzed 94 patients who underwent hepatic resection and intra-operative ablation of metastatic NETs and found that overall survival was 80% at 5 years. Notably, a Ki-67 index ≥ 3.5 was a significant predictor of decreased survival [135]. Jensen and colleagues evaluated biomarkers that were predictive of outcome after cytoreductive surgery on functional carcinoid tumors that had metastasized to the liver. The authors found that a reduction of CgA ≥ 80% was highly predictive of complete resolution of symptoms and disease stabilization. Urinary 5-HIAA was also evaluated; patients reported significant symptom relief when levels reduced ≥ 80% or normalized [152].

Combined surgery and neoadjuvant therapy have also been described in the treatment of patients with metastatic NETs. In a large retrospective series of 4892 patients with localized PNEts, Xie and colleagues evaluated outcomes of patients who received perioperative systemic therapy with those who received surgery alone. In a matched cohort analysis, the perioperative systemic therapy group had a significantly shorter overall survival compared to the surgery alone group; of note, the median overall survival was not reached in either cohort [153]. Some studies have shown good or improved overall survival with the addition of peri-operative chemotherapy [154,155,156,157]. In a study evaluating patients who underwent R0/R1 resection of metastatic PNETs, those patients with neuroendocrine liver metastases who received preoperative fluorouracil, doxorubicin, and streptozocin had significantly higher overall survival (median 97.3 months vs. 65.0 months) and recurrence-free survival (median 24.8 months vs. 12.1 months) compared to those patients who did not receive preoperative chemotherapy [155]. Notably, none of the aforementioned studies evaluated the impact of outcomes related to biomarkers. Data on combination therapies used to downsize NETs to allow for curative resection remain limited and there is marked heterogeneity in the available studies; this remains a field for further development.

## 6. Conclusions

NETs represent a heterogeneous group of neoplasms; however, the presence of liver metastases universally portends worse patient outcomes. The disease is often diagnosed at a later stage, when patients present with debilitating hormonal symptoms. The evaluation and understanding of widely available biomarkers such as CgA, 5-HIAA, and Ki-67 have allowed for more accurate prognostication of survival and treatment response. Additionally, newer tests such as the NETest and genomic sequencing are allowing clinicians and researchers to better understand, diagnose, and treat these tumors. The application of these biomarkers and tests to better tailor treatment decisions has the potential to provide more efficacious treatment options for patients. Despite these advances, there remains an ongoing need to better understand the genetic aberrations and biomarkers that can allow for earlier and accurate disease diagnosis, predict prognosis, and allow for personalized treatment options. One of the key recommendations that arose from the NET Task Force of the National Cancer Institute GI Steering Committee was to determine the association between molecular subtypes and treatment effects of specific drugs [70]. Many researchers are addressing this task, with newer studies evaluating prognostic markers that impact treatment outcomes. Multiple parallel avenues of research are focused on better characterizing epigenetic changes in neuroendocrine tumors that may serve as more useful biomarkers, including DNA methylation, histone modification, chromosome shattering, and the tumor microenvironment [158,159,160,161]. This research has the potential to further elucidate the factors that drive NET progression and to better assess and treat patients living with the disease.

## Figures and Tables

**Table 1:** The World Health Organization (WHO) 2022 Classification of Neuroendocrine Neoplasms [3,4].

Neuroendocrine Neoplasm	Classification	Mitotic Rate (Mitoses/2 mm^2^)	Ki-67 Index
**Gastroenteropancreatic** Well-differentiated NET	NET, grade 1	<2	<3%
	NET, grade 2	2–20	3–20%
	NET, grade 3	>20	>20%
**Upper aerodigestive tract and salivary glands** Well-differentiated NET	NET, grade 1	<2	<20%
	NET, grade 2	2–10	<20%
	NET, grade 3	>10	>20%
**Lung and thymus** Well-differentiated NET	Typical carcinoid, NET, grade 1	<2	
	Atypical carcinoid, NET, grade 2	2–10	
	Carcinoids/NETs with elevated mitotic counts and/or Ki-67 index	>10	>30%
